# Are auditory verbal hallucinations in schizophrenia just “voices” or something different?: Clinical, empirical and phenomenological perspectives

**DOI:** 10.1192/j.eurpsy.2023.1288

**Published:** 2023-07-19

**Authors:** A. U. Parnas

**Affiliations:** Psychiatry, University Health Centre Amager, Copenhagen, Denmark

## Abstract

**Introduction:**

Auditory verbal hallucinations (AVH) form a central symptom in the current diagnosis of schizophrenia in the DSM-5 and ICD-10. In both internatinal classifications, hallucinations are considered an erroneous perception without external stimulation. AVH are often viewed as a well-defined entity in itself with certain quasi objective properties. They seem not to arise from nothing but are preceded and accompanied by the anomalies of subjective experiences such as e.g. feeling different, thought aloud and thought interference and experiences difficult to distinguish these phenomena from “hearing voices”. Several recent reviews point to the complexity of the nature of AVH and advocate the involvement of contextual issues and co-occurring psychopathology.

**Objectives:**

The aims of this study were to examine the qualitative aspects of the experience of hearing voices, the period of disclosure of AVH and the concomitant subjective experiences (self-disorders) in a group of readmitted patients with a diagnosis of paranoid schizophrenia and experiencing AVH.

**Methods:**

We performed an empirical qualitative and phenomenologically oriented investigation of the experiential and existential aspects of AVH. Twenty patients with AVH and fulfilling the ICD-10 criteria of schizophrenia were interviewed with semi-structured questionnaire, covering the aims of this study. The interview encouraged the patients to reflect and express themselves freely. We used 26 items (domains stream of consciousness and basic self ) from the Examination of Anomalous Self-Experience (EASE).

**Results:**

The disclosure of AVH happened when the patient arrived at a situation of subjective suffering or dysfunction in life, often several years after their beginning. Several participants were not able to determine whether voices were in the “internal” or “external” space. They did not consider their AVH as being analogous to a perception of an external object. The patients were continuously in doubt whether their experiences merited the name of voices or merely thoughts. The terminological status of the AVH as “voices” was typically acquired in the psychiatric setting.

**Conclusions:**

AVH themselves are not a sufficient sign of mental disturbance unless it is an aspect of a profound change in the structure of consciousness. There is an apparent continuity between thinking and hallucinations. AVH articulate themselves within the intimidate sphere of the patient in another dimension and not in the shared social world as a real perception. The patient’s difficulties to describe the detailed features of hallucinations could be an expression of the psychiatrist‘s insistence on framing the hallucination in the perceptual space to which it does not belong leading to a risk of missing the phenomenon.

**Disclosure of Interest:**

None Declared

